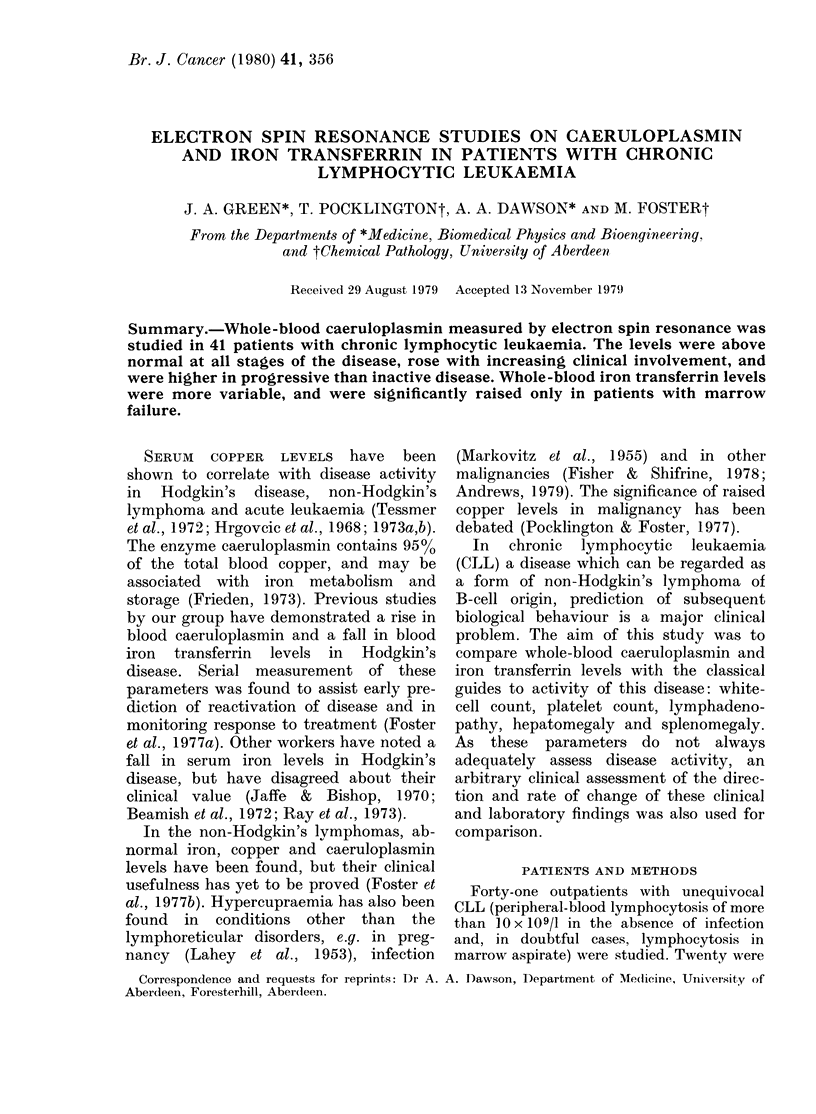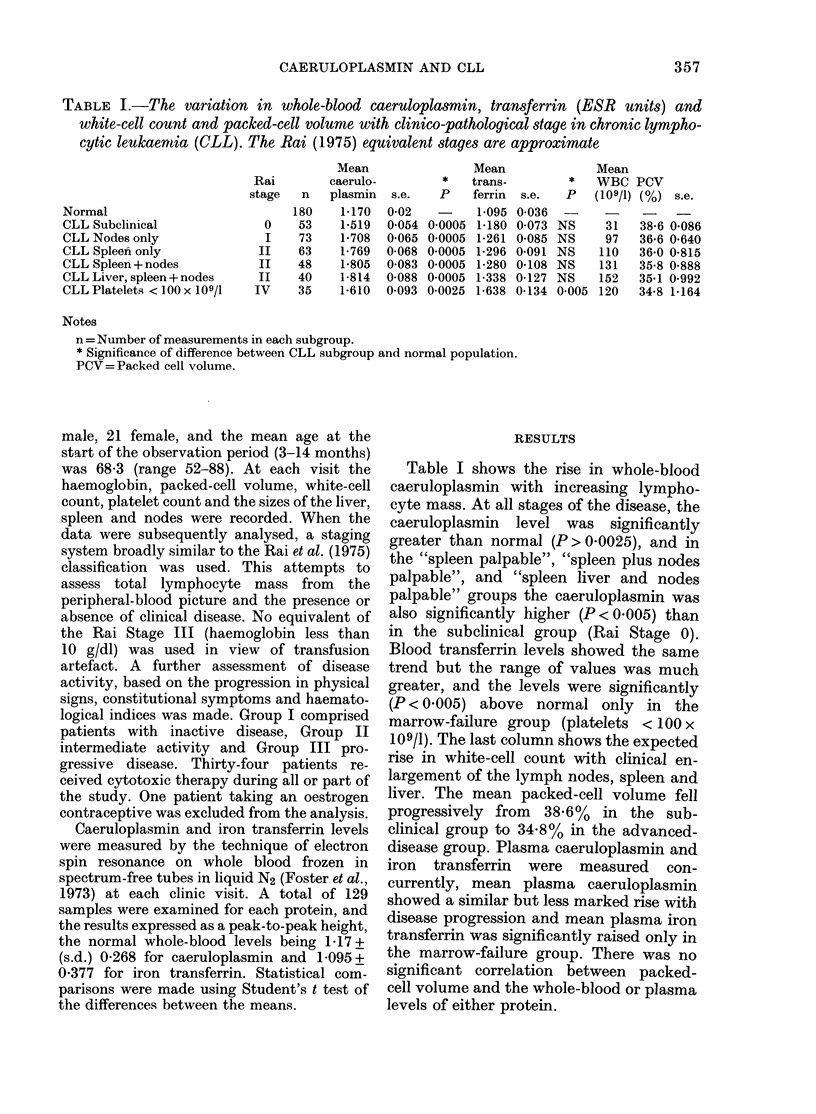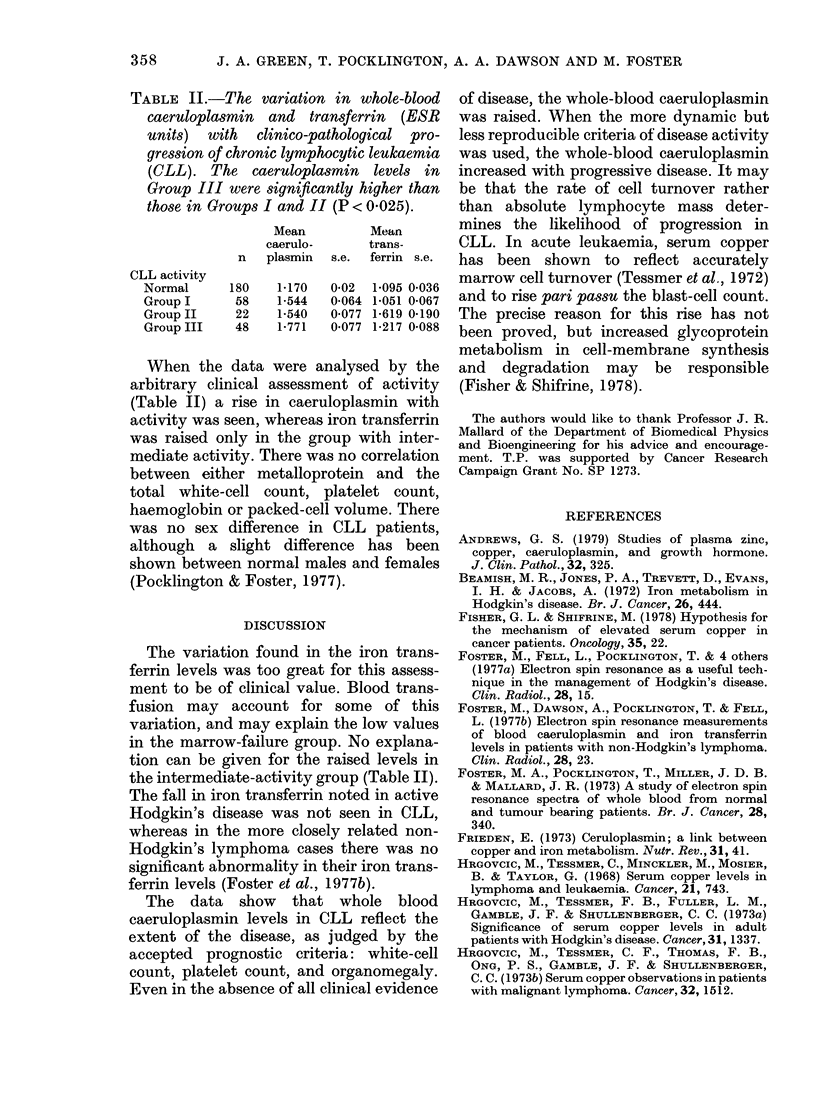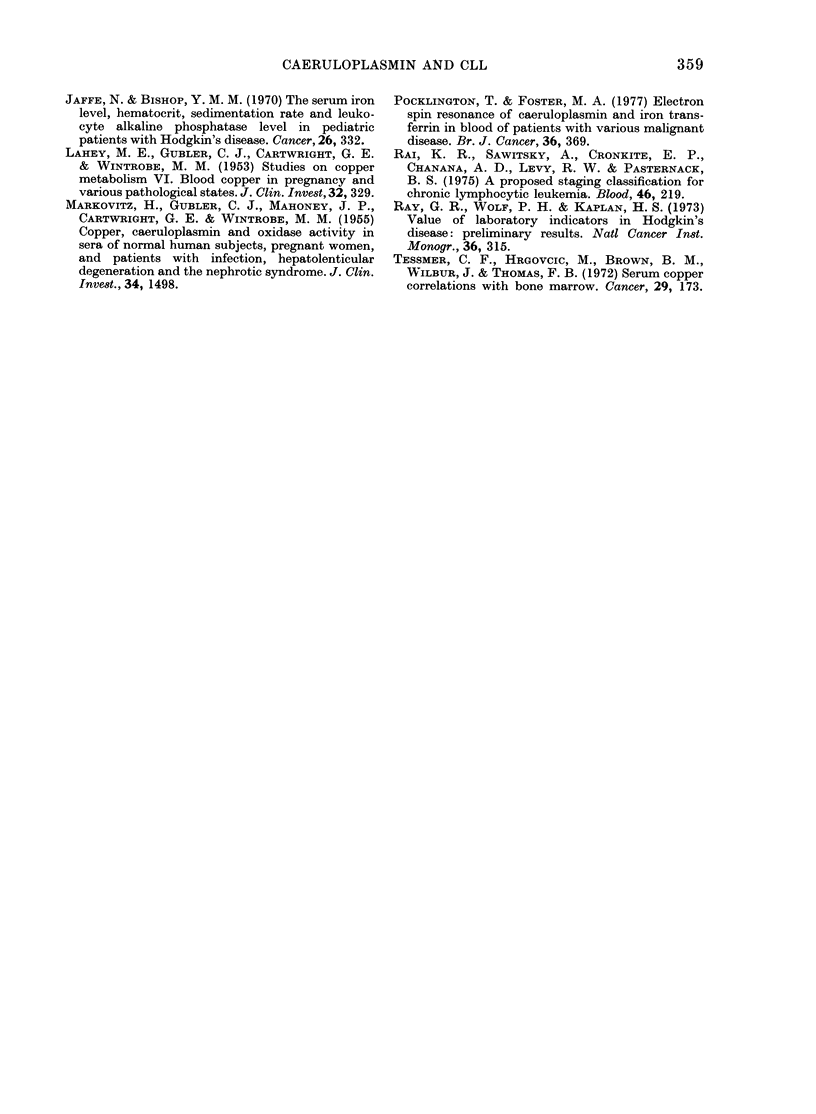# Electron spin resonance studies on caeruloplasmin and iron transferrin in patients with chronic lymphocytic leukaemia.

**DOI:** 10.1038/bjc.1980.58

**Published:** 1980-03

**Authors:** J. A. Green, T. Pocklington, A. A. Dawson, M. Foster

## Abstract

Whole-blood caeruloplasmin measured by electron spin resonance was studied in 41 patients with chronic lymphocytic leukaemia. The levels were above normal at all stages of the disease, rose with increasing clinical involvement, and were higher in progressive than inactive disease. Whole-blood iron transferrin levels were more variable, and were significantly raised only in patients with marrow failure.


*Br. J. Cancer* (1980) **41**, 356

### ELECTRON SPIN RESONANCE STUDIES ON CAERULOPLASMIN AND IRON TRANSFERRIN IN PATIENTS WITH CHRONIC LYMPHOCYTIC LEUKAEMIA

J. A. GREEN*, T. POCKLINGTON†, A. A. DAWSON* AND M. FOSTER†

*From the Departments of *Medicine, Biomedical Physics and Bioengineering, and †Chemical Pathology, University of Aberdeen*

Received 29 August 1979 Accepted 13 November 1979

**Summary.—Whole-blood caeruloplasmin measured by electron spin resonance was studied in 41 patients with chronic lymphocytic leukaemia. The levels were above normal at all stages of the disease, rose with increasing clinical involvement, and were higher in progressive than inactive disease. Whole-blood iron transferrin levels were more variable, and were significantly raised only in patients with marrow failure.**

SERUM COPPER LEVELS have been shown to correlate with disease activity in Hodgkin's disease, non-Hodgkin's lymphoma and acute leukaemia (Tessmer *et al.*, 1972; Hrgovcic *et al.*, 1968; 1973*a,b*). The enzyme caeruloplasmin contains 95% of the total blood copper, and may be associated with iron metabolism and storage (Frieden, 1973). Previous studies by our group have demonstrated a rise in blood caeruloplasmin and a fall in blood iron transferrin levels in Hodgkin's disease. Serial measurement of these parameters was found to assist early prediction of reactivation of disease and in monitoring response to treatment (Foster *et al.*, 1977*a*). Other workers have noted a fall in serum iron levels in Hodgkin's disease, but have disagreed about their clinical value (Jaffe & Bishop, 1970; Beamish *et al.*, 1972; Ray *et al.*, 1973).

In the non-Hodgkin's lymphomas, abnormal iron, copper and caeruloplasmin levels have been found, but their clinical usefulness has yet to be proved (Foster *et al.*, 1977*b*). Hypercupraemia has also been found in conditions other than the lymphoreticular disorders, *e.g.* in pregnancy (Lahey *et al.*, 1953), infection (Markovitz *et al.*, 1955) and in other malignancies (Fisher & Shifrine, 1978; Andrews, 1979). The significance of raised copper levels in malignancy has been debated (Pocklington & Foster, 1977).

In chronic lymphocytic leukaemia (CLL) a disease which can be regarded as a form of non-Hodgkin's lymphoma of B-cell origin, prediction of subsequent biological behaviour is a major clinical problem. The aim of this study was to compare whole-blood caeruloplasmin and iron transferrin levels with the classical guides to activity of this disease: white-cell count, platelet count, lymphadenopathy, hepatomegaly and splenomegaly. As these parameters do not always adequately assess disease activity, an arbitrary clinical assessment of the direction and rate of change of these clinical and laboratory findings was also used for comparison.

### PATIENTS AND METHODS

Forty-one outpatients with unequivocal CLL (peripheral-blood lymphocytosis of more than $10 \times 10^9$/l in the absence of infection and, in doubtful cases, lymphocytosis in marrow aspirate) were studied. Twenty were

Correspondence and requests for reprints: Dr A. A. Dawson, Department of Medicine, University of Aberdeen, Foresterhill, Aberdeen.

TABLE I.—*The variation in whole-blood caeruloplasmin, transferrin (ESR units) and white-cell count and packed-cell volume with clinico-pathological stage in chronic lymphocytic leukaemia (CLL). The Rai (1975) equivalent stages are approximate*

| | Rai stage | n | Mean caeruloplasmin | s.e. | * *P* | Mean transferrin | s.e. | * *P* | Mean WBC ($10^9$/l) | PCV (%) | s.e. |
|---|---|---|---|---|---|---|---|---|---|---|---|
| Normal | | 180 | 1·170 | 0·02 | — | 1·095 | 0·036 | — | — | — | — |
| CLL Subclinical | 0 | 53 | 1·519 | 0·054 | 0·0005 | 1·180 | 0·073 | NS | 31 | 38·6 | 0·086 |
| CLL Nodes only | I | 73 | 1·708 | 0·065 | 0·0005 | 1·261 | 0·085 | NS | 97 | 36·6 | 0·640 |
| CLL Spleen only | II | 63 | 1·769 | 0·068 | 0·0005 | 1·296 | 0·091 | NS | 110 | 36·0 | 0·815 |
| CLL Spleen + nodes | II | 48 | 1·805 | 0·083 | 0·0005 | 1·280 | 0·108 | NS | 131 | 35·8 | 0·888 |
| CLL Liver, spleen + nodes | II | 40 | 1·814 | 0·088 | 0·0005 | 1·338 | 0·127 | NS | 152 | 35·1 | 0·992 |
| CLL Platelets $< 100 \times 10^9$/l | IV | 35 | 1·610 | 0·093 | 0·0025 | 1·638 | 0·134 | 0·005 | 120 | 34·8 | 1·164 |

Notes n = Number of measurements in each subgroup.
* Significance of difference between CLL subgroup and normal population.
PCV = Packed cell volume.

male, 21 female, and the mean age at the start of the observation period (3–14 months) was 68·3 (range 52–88). At each visit the haemoglobin, packed-cell volume, white-cell count, platelet count and the sizes of the liver, spleen and nodes were recorded. When the data were subsequently analysed, a staging system broadly similar to the Rai *et al.* (1975) classification was used. This attempts to assess total lymphocyte mass from the peripheral-blood picture and the presence or absence of clinical disease. No equivalent of the Rai Stage III (haemoglobin less than 10 g/dl) was used in view of transfusion artefact. A further assessment of disease activity, based on the progression in physical signs, constitutional symptoms and haematological indices was made. Group I comprised patients with inactive disease, Group II intermediate activity and Group III progressive disease. Thirty-four patients received cytotoxic therapy during all or part of the study. One patient taking an oestrogen contraceptive was excluded from the analysis.

Caeruloplasmin and iron transferrin levels were measured by the technique of electron spin resonance on whole blood frozen in spectrum-free tubes in liquid $N_2$ (Foster *et al.*, 1973) at each clinic visit. A total of 129 samples were examined for each protein, and the results expressed as a peak-to-peak height, the normal whole-blood levels being $1{\cdot}17 \pm$ (s.d.) 0·268 for caeruloplasmin and $1{\cdot}095 \pm 0{\cdot}377$ for iron transferrin. Statistical comparisons were made using Student's *t* test of the differences between the means.

### RESULTS

Table I shows the rise in whole-blood caeruloplasmin with increasing lymphocyte mass. At all stages of the disease, the caeruloplasmin level was significantly greater than normal ($P > 0{\cdot}0025$), and in the "spleen palpable", "spleen plus nodes palpable", and "spleen liver and nodes palpable" groups the caeruloplasmin was also significantly higher ($P < 0{\cdot}005$) than in the subclinical group (Rai Stage 0). Blood transferrin levels showed the same trend but the range of values was much greater, and the levels were significantly ($P < 0{\cdot}005$) above normal only in the marrow-failure group (platelets $< 100 \times 10^9$/l). The last column shows the expected rise in white-cell count with clinical enlargement of the lymph nodes, spleen and liver. The mean packed-cell volume fell progressively from 38·6% in the subclinical group to 34·8% in the advanced-disease group. Plasma caeruloplasmin and iron transferrin were measured concurrently, mean plasma caeruloplasmin showed a similar but less marked rise with disease progression and mean plasma iron transferrin was significantly raised only in the marrow-failure group. There was no significant correlation between packed-cell volume and the whole-blood or plasma levels of either protein.

TABLE II.—*The variation in whole-blood caeruloplasmin and transferrin (ESR units) with clinico-pathological progression of chronic lymphocytic leukaemia (CLL). The caeruloplasmin levels in Group III were significantly higher than those in Groups I and II* ($P < 0{\cdot}025$).

| | n | Mean caeruloplasmin | s.e. | Mean transferrin | s.e. |
|---|---|---|---|---|---|
| CLL activity | | | | | |
| Normal | 180 | 1·170 | 0·02 | 1·095 | 0·036 |
| Group I | 58 | 1·544 | 0·064 | 1·051 | 0·067 |
| Group II | 22 | 1·540 | 0·077 | 1·619 | 0·190 |
| Group III | 48 | 1·771 | 0·077 | 1·217 | 0·088 |

When the data were analysed by the arbitrary clinical assessment of activity (Table II) a rise in caeruloplasmin with activity was seen, whereas iron transferrin was raised only in the group with intermediate activity. There was no correlation between either metalloprotein and the total white-cell count, platelet count, haemoglobin or packed-cell volume. There was no sex difference in CLL patients, although a slight difference has been shown between normal males and females (Pocklington & Foster, 1977).

### DISCUSSION

The variation found in the iron transferrin levels was too great for this assessment to be of clinical value. Blood transfusion may account for some of this variation, and may explain the low values in the marrow-failure group. No explanation can be given for the raised levels in the intermediate-activity group (Table II). The fall in iron transferrin noted in active Hodgkin's disease was not seen in CLL, whereas in the more closely related non-Hodgkin's lymphoma cases there was no significant abnormality in their iron transferrin levels (Foster *et al.*, 1977*b*).

The data show that whole blood caeruloplasmin levels in CLL reflect the extent of the disease, as judged by the accepted prognostic criteria: white-cell count, platelet count, and organomegaly. Even in the absence of all clinical evidence of disease, the whole-blood caeruloplasmin was raised. When the more dynamic but less reproducible criteria of disease activity was used, the whole-blood caeruloplasmin increased with progressive disease. It may be that the rate of cell turnover rather than absolute lymphocyte mass determines the likelihood of progression in CLL. In acute leukaemia, serum copper has been shown to reflect accurately marrow cell turnover (Tessmer *et al.*, 1972) and to rise *pari passu* the blast-cell count. The precise reason for this rise has not been proved, but increased glycoprotein metabolism in cell-membrane synthesis and degradation may be responsible (Fisher & Shifrine, 1978).

The authors would like to thank Professor J. R. Mallard of the Department of Biomedical Physics and Bioengineering for his advice and encouragement. T.P. was supported by Cancer Research Campaign Grant No. SP 1273.

### REFERENCES

ANDREWS, G. S. (1979) Studies of plasma zinc, copper, caeruloplasmin, and growth hormone. *J. Clin. Pathol.*, **32**, 325.

BEAMISH, M. R., JONES, P. A., TREVETT, D., EVANS, I. H. & JACOBS, A. (1972) Iron metabolism in Hodgkin's disease. *Br. J. Cancer*, **26**, 444.

FISHER, G. L. & SHIFRINE, M. (1978) Hypothesis for the mechanism of elevated serum copper in cancer patients. *Oncology*, **35**, 22.

FOSTER, M., FELL, L., POCKLINGTON, T. & 4 others (1977*a*) Electron spin resonance as a useful technique in the management of Hodgkin's disease. *Clin. Radiol.*, **28**, 15.

FOSTER, M., DAWSON, A., POCKLINGTON, T. & FELL, L. (1977*b*) Electron spin resonance measurements of blood caeruloplasmin and iron transferrin levels in patients with non-Hodgkin's lymphoma. *Clin. Radiol.*, **28**, 23.

FOSTER, M. A., POCKLINGTON, T., MILLER, J. D. B. & MALLARD, J. R. (1973) A study of electron spin resonance spectra of whole blood from normal and tumour bearing patients. *Br. J. Cancer*, **28**, 340.

FRIEDEN, E. (1973) Ceruloplasmin; a link between copper and iron metabolism. *Nutr. Rev.*, **31**, 41.

HRGOVCIC, M., TESSMER, C., MINCKLER, M., MOSIER, B. & TAYLOR, G. (1968) Serum copper levels in lymphoma and leukaemia. *Cancer*, **21**, 743.

HRGOVCIC, M., TESSMER, F. B., FULLER, L. M., GAMBLE, J. F. & SHULLENBERGER, C. C. (1973*a*) Significance of serum copper levels in adult patients with Hodgkin's disease. *Cancer*, **31**, 1337.

HRGOVCIC, M., TESSMER, C. F., THOMAS, F. B., ONG, P. S., GAMBLE, J. F. & SHULLENBERGER, C. C. (1973*b*) Serum copper observations in patients with malignant lymphoma. *Cancer*, **32**, 1512.

JAFFE, N. & BISHOP, Y. M. M. (1970) The serum iron level, hematocrit, sedimentation rate and leukocyte alkaline phosphatase level in pediatric patients with Hodgkin's disease. *Cancer*, **26**, 332.

LAHEY, M. E., GUBLER, C. J., CARTWRIGHT, G. E. & WINTROBE, M. M. (1953) Studies on copper metabolism VI. Blood copper in pregnancy and various pathological states. *J. Clin. Invest*, **32**, 329.

MARKOVITZ, H., GUBLER, C. J., MAHONEY, J. P., CARTWRIGHT, G. E. & WINTROBE, M. M. (1955) Copper, caeruloplasmin and oxidase activity in sera of normal human subjects, pregnant women, and patients with infection, hepatolenticular degeneration and the nephrotic syndrome. *J. Clin. Invest.*, **34**, 1498.

POCKLINGTON, T. & FOSTER, M. A. (1977) Electron spin resonance of caeruloplasmin and iron transferrin in blood of patients with various malignant disease. *Br. J. Cancer*, **36**, 369.

RAI, K. R., SAWITSKY, A., CRONKITE, E. P., CHANANA, A. D., LEVY, R. W. & PASTERNACK, B. S. (1975) A proposed staging classification for chronic lymphocytic leukemia. *Blood*, **46**, 219.

RAY, G. R., WOLF, P. H. & KAPLAN, H. S. (1973) Value of laboratory indicators in Hodgkin's disease: preliminary results. *Natl Cancer Inst. Monogr.*, **36**, 315.

TESSMER, C. F., HRGOVCIC, M., BROWN, B. M., WILBUR, J. & THOMAS, F. B. (1972) Serum copper correlations with bone marrow. *Cancer*, **29**, 173.